# Factors associated with delayed initiation and non-exclusive breastfeeding among children in India: evidence from national family health survey 2019-21

**DOI:** 10.1186/s13006-023-00566-0

**Published:** 2023-06-06

**Authors:** Madhurima Sharma, Abhishek Anand, Indrajit Goswami, Manas Ranjan Pradhan

**Affiliations:** International Institute for Population Sciences, Govandi Station Road, Deonar, Mumbai, 400088 India

**Keywords:** Infant, Delayed breastfeeding, Non-exclusive breastfeeding, Logistic regression, India

## Abstract

**Background:**

In India, more than half of the newborns experience delayed breastfeeding, and non-exclusive breastfeeding is practiced in 63% of babies below the age of six months. The goal of this study is to investigate the extent to which external environment, demographic and socioeconomic, pregnancy and birthing characteristics, as well as utilization of maternal care services, are associated with delayed initiation and non-exclusive breastfeeding among children in India.

**Methods:**

Data was gathered from the fifth round of the National Family Health Survey (NFHS), which was conducted in 2019-21. This study used information on 85,037 singleton infants aged 0–23 months and 22,750 singleton infants aged 0–5 months. Delayed initiation of breastfeeding and non-exclusive breastfeeding was used as outcome variables in this study. Unadjusted and adjusted multivariable binary logistic regression was performed to analyse the association of delayed breastfeeding and non-exclusive breastfeeding with selected background characteristics.

**Results:**

Factors significantly associated with increased risks of delayed initiation of breastfeeding included infants from in the central region (OR 2.19; 95% CI 2.09, 2.29), mothers in the 20 to 29 years age group at the time of childbirth (OR 1.02; 95% CI 0.98, 1.05), caesarean deliveries (OR 1.97; 95% CI 1.90, 2.05). The likelihoods for non-exclusive breastfeeding significantly increased among children belonging to the richest household status (OR 1.30; 95% CI 1.17, 1.45), mothers who had less than nine months of pregnancy period (OR 1.15; 95% CI 1.06, 1.25), and mothers who gave birth in non-health facility (OR 1.17; 95% CI 1.05, 1.31).

**Conclusions:**

The connections between several different categories of factors and non-exclusive breastfeeding and delayed breastfeeding initiation show the need for comprehensive public health programmes using a multi-sectoral approach to promote breastfeeding behaviours in India.

## Background

Breastfeeding plays a significant role in promoting child health due to its significant health advantages for both children and mothers [1]. Breastfeeding practices are subject to influence by a multitude of factors such as cultural, socioeconomic, and individual aspects associated with both the infant and the mother [2, 3]. Studies from developing nations demonstrate that a longer nursing period is linked to a child’s greater linear growth [4, 5]. Additionally, recent research indicates that breastfeeding for a longer duration is also beneficial for mothers as it reduces the chance of developing type I diabetes and being overweight later in life [6, 7]. However, many countries still have low rates of appropriate breastfeeding practices [8]. Sub optimal breastfeeding practices result in the loss of 117 million years of life in developing nations [9]. In order to achieve the best possible growth, development, and health, the World Health Organization (WHO) and United Nations Children’s Fund (UNICEF) advised in 2003 that breastfeeding be initiated as soon as possible after birth and continued exclusively for six months [10, 11].

Early initiation of breastfeeding, within one hour of birth, protects the newborn from acquiring infection and reduces newborn mortality also, a newborn should only be breastfed for the first six months of life, according to WHO and Indian government guidelines [12, 13].

Additionally, they recommend starting supplementary feeding at six months of age and continuing frequent, on-demand breastfeeding for at least two years. The advice to continue breastfeeding is based on the fact that breast milk continues to be crucial for a child’s growth, development, and nutrition even after they become six months old. Studies from India and other countries report that breastfeeding duration is influenced by a number of different factors. Maternal factors that have been shown to be associated with the duration of breastfeeding include age at birth, educational status, infant feeding attitudes, occupation or work status, and smoking habits [14–16].

Several studies have been conducted in developing countries, including India, to examine the determinants of early initiation and exclusive breastfeeding [17–20]. According to recent estimates, only around two-fifths (43%) of children born in the six months preceding the survey were breastfed within one hour of delivery, indicating that more than half (57%) of them had delayed breastfeeding. In India, 63% of infants under the age of six months are non-exclusively breastfed. Many children under the age of six months consume other liquids, such as plain water (10%), other milk (8%), or complementary foods (11%), in addition to breastmilk [21].

The government of India initiated a programme ‘Mothers Absolute Affection’ (MAA), for the promotion of breastfeeding in 2016. However, despite the substantial efforts by healthcare practitioners in India to encourage breastfeeding practice, the country’s breastfeeding rate remains below the WHO’s targeted objective of 50%. To the best of our knowledge, no well-documented national-level analysis has been conducted in India concerning poor breastfeeding habits, such as delayed initiation and non-exclusive breastfeeding among children. Thus, the aim of this study is to investigate the extent to which external environment, demographic and socioeconomic, pregnancy and birthing characteristics, as well as utilization of maternal care services, are associated with delayed initiation and non-exclusive breastfeeding among children in India.

## Methods

### Data

We used data from the fifth round of the National Family Health Survey (NFHS-5), which was conducted in 2019-21 under the aegis of the Ministry of Health and Family Welfare (MoHFW), India. NFHS-5 is a nationally representative large-scale survey which covers all states and union territories of India. The survey covered 636,699 households, 724,115 women, and 101,839 men. The prime objective of NFHS is to provide reliable data on various issues related to health and family welfare, such as fertility, mortality, maternal and child health, sexual behaviours, domestic violence etc. The NFHS-5 employed a stratified two-stage stratified random sampling design for data collection (29). This study used information on 85,037 singleton infants aged 0–23 months and 22,750 singleton infants aged 0–5 months. This research was carried out in conformity with internationally agreed-upon ethical norms for medical research. As this is a secondary analysis of NFHS data that is available in the public domain, no ethical approval is necessary [22].

### Variables

#### Outcome variables

The first outcome variable in this study is for infants aged 0–23 months, “Delayed initiation of breastfeeding”, i.e., infants who were put to breast one hour or more after delivery. This is based on the question asked to mothers, “*How long after birth did you start breastfeeding?*”

The second outcome variable is “Non-exclusive breastfeeding”, for infants aged 0–5 months. On the basis of the existing literature on Exclusive breastfeeding (EBF), exclusive breastfeeding is measured as infants aged 0 to 5 months who were breastfed on the day prior to a survey administered to their mothers and received no other type of food or fluid [23]. This outcome variable was based on a combination of questions, “*Are you still breastfeeding?*” and “*Did the child drink or eat anything other than breastmilk yesterday during the day or at night (last 24 hours)?*”

#### Exposure variables

This study included relevant exposure variables suggested by existing literature [19, 24]. The variables were categorized into demographic and socioeconomic characteristics, pregnancy and birth-related characteristics, maternal health service utilization and external environment factors. The demographic and socioeconomic characteristics include the current age of mothers (15–24 years, 24–34 years and 35 + years), maternal age at childbirth (less than 20 years, 20–29 years, 30–39 years, 40 + years), religion (Hindu, Muslim, Christian, Others), caste (Scheduled Castes – SC, Scheduled Tribes- ST, Other Backward Classes – OBC, others), maternal education (no education, primary, secondary, higher), marital status (currently married, formerly married), mass media exposure (not exposed, exposed).

The pregnancy and birth-related characteristics include the desire of pregnancy (wanted then, wanted later/ no more wanted), delivery complications (no, yes), duration of pregnancy (nine months and above, less than nine months), size of child at birth (average size, smaller than average size, larger than average size), sex of the child (male, female). The maternal health service utilization factors include the number of ANC visits (4 + times, 1–3 times, none), birth attendants (none/untrained delivery attendants, trained delivery attendants), place of delivery (institutional delivery, non-institutional delivery), mode of delivery (non-caesarean section, caesarean section), postnatal care services (no check, doctor, nurse/midwife, non-skilled provider). The external environmental factors include region (north, central, east, northeast, west, south), and place of residence (urban, rural).

### Statistical analysis

Descriptive statistics were obtained, and chi-square analysis was used to assess the association of selected background characteristics with the outcome variable of interest. Additionally, the unadjusted and adjusted multivariable binary logistic regression was performed to analyse the association of delayed breastfeeding and non-exclusive breastfeeding with selected background characteristics. The baseline model of the multivariate analysis included all the potential factors along with the outcome variable. The backward elimination approach was then utilized to keep those variables that were significantly related to outcome variables. The statistical analysis was done in Stata v16.0, and a significance level of *p* < 0.05 was used in the analysis.

## Results

### Sample profile

The detailed demographic, socioeconomic, pregnancy and birth-related factors, and maternal health service utilization determinants of children aged 0–5 months and 0–23 months included in our research are presented in Table 1. Around 28% and 27% of children aged 0–23 months were from central and eastern regions, respectively. About 30% of children aged 0–5 months were from the central region. More than half of the children were from rural regions. Approximately 79% of the children were born to mothers who followed the Hindu religion. Almost 70% of the children were born to mothers who had secondary and above level of education. Around 57% of the children were born to mothers who had four or more antenatal visits. More than 90% of deliveries were institutional delivery.

**Table 1:** Demographic, socioeconomic, pregnancy and birth-related characteristics and maternal health service utilization determinants of children aged 0–23 months and 0–5 months in India, 2019-21

Variables	Children aged 0–23 months (*n* = 85,037)		Children aged 0–5 months (*n* = 22,750)						
	*n*	Distribution (%)	0–1 month			2–3 months		4–5 months	
			*n*	Distribution (%)		*n*	Distribution (%)	*n*	Distribution (%)
**External environment factors**									
**Region**									
North	11,269	13.25	1,098	15.18	1,204		15.39	1,124	14.62
Central	23,746	27.92	2,107	29.13	2,367		30.24	2,422	31.5
East	22,328	26.26	1,851	25.59	1,941		24.81	1,862	24.21
Northeast	3,016	3.55	262	3.62	254		3.25	247	3.21
West	10,390	12.22	844	11.66	789		10.08	806	10.48
South	14,288	16.8	1,072	14.82	1,271		16.23	1,229	15.98
**Place of residence**									
Urban	22,096	25.98	1,724	23.84	1,876		23.98	1,974	25.67
Rural	62,941	74.02	5,510	76.16	5,950		76.02	5,716	74.33
**Demographic & socioeconomic characteristics**									
**Maternal age at childbirth (years)**									
Less than 20	26,683	31.38	2,157	29.81		2,243	28.66	2,299	29.9
20–29	55,634	65.42	4,803	66.4		5,342	68.26	5,153	67.01
30+	2,719	3.2	274	3.79		242	3.09	237	3.08
**Religion**									
Hindu	67,683	79.59	5,800	80.18		6,244	79.78	6,014	78.21
Others	17,354	20.41	1,434	19.82		1,582	20.22	1,676	21.79
**Caste of mother**									
SC	19,722	23.19	1,744	24.1		1,872	23.93	1,756	22.83
ST	8,671	10.2	774	10.7		806	10.29	776	10.09
OBC	37,202	43.75	3,175	43.88		3,471	44.35	3,377	43.91
Others	19,442	22.86	1,542	21.32		1,677	21.43	1,782	23.17
**Wealth index**									
Poorest	20,252	23.82	1,796	24.82		1,821	23.27	1,848	24.03
Poor	18,353	21.58	1,683	23.26		1,675	21.4	1,659	21.57
Middle	16,942	19.92	1,400	19.35		1,600	20.44	1,477	19.21
Richer	15,878	18.67	1,344	18.58		1,532	19.58	1,402	18.23
Richest	13,612	16.01	1,011	13.98		1,198	15.31	1,304	16.95
**Maternal education level**									
No education	15,788	18.57	4,160	18.29		1,323	16.91	1,415	18.4
Primary	9,515	11.19	2,536	11.15		829	10.59	885	11.51
Secondary and above	59,733	70.24	16,053	70.56		5,674	72.5	5,389	70.08
**Maternal mass-media exposure**									
Not Exposed	23,888	28.09	6,447	28.34		2,191	28	2,115	27.51
Exposed	61,149	71.91	16,303	71.66		5,635	72	5,575	72.49
**Pregnancy & birth-related characteristics**									
**Desire of pregnancy**									
Wanted then	78,117	91.86	20,746	91.19		7,139	91.22	7,032	91.44
Wanted later/ no more wanted	6,920	8.14	2,004	8.81		687	8.78	658	8.56
**Preterm birth**									
No	74,604	87.73	19,825	87.14		6,836	87.35	6,709	87.24
Yes	10,433	12.27	2,925	12.86		990	12.65	981	12.76
**Sex of the child**									
Male	43,911	51.64	11,681	51.34		4,056	51.83	3,933	51.14
Female	41,126	48.36	11,069	48.66		3,770	48.17	3,757	48.86
**Maternal health service utilization**									
**Number of ANC**									
4 + times	48,211	56.69	13,136	57.74		4,547	58.1	4,332	56.33
1–3 times	28,597	33.63	8,081	35.52		2,740	35.01	2,807	36.51
None	8,229	9.68	1,533	6.74		539	6.89	551	7.16
**Place of delivery**									
Institutional delivery	77,144	90.72	6,534	90.32		7,130	91.11	6,980	90.76
Delivery at home	7,893	9.28	700	9.68		696	8.89	710	9.24
**Mode of delivery**									
Non-Caesarean section	64,919	76.34	17,318	76.12		5,963	76.2	5,741	74.66
Caesarean section	20,118	23.66	5,432	23.88		1,863	23.8	1,949	25.34
**Postnatal care services**									
No check	14,886	17.51	3,954	17.38		1,344	17.18	1,288	16.75
Doctor	43,125	50.71	11,318	49.75		3,964	50.66	3,828	49.78
Nurse/Midwife	24,561	28.88	6,798	29.88		2,291	29.27	2,346	30.5
Non-skilled provider	2,464	2.9	680	2.99		227	2.9	228	2.97
**Total**	**100**	**85,037**	**100**	**7,234**		**100**	**7,826**	**100**	**7,690**

*Note: All values are weighted by the sampling probability*

### Delayed initiation of breastfeeding and non-exclusive breastfeeding by background characteristics

Prevalence and association of delayed initiation of breastfeeding and non-exclusive breastfeeding with some selected background characteristics among children aged 0–23 months and 0–5 months are presented in Table 2. Factors significantly associated with delayed initiation of breastfeeding included children from different regions, maternal age at childbirth (years), religion, caste, maternal education level, duration of pregnancy and mode of delivery (*p* < 0.001). Delayed initiation of breastfeeding was also associated with the place of delivery. The prevalence of delayed initiation of breastfeeding among infants aged 0–23 months in India is 57%. In the central area, the prevalence of delayed breastfeeding is about 70%. In the case of uneducated mothers, the estimated prevalence of delayed breastfeeding initiation is 61%. It is higher than those who had a certain level of education. For mothers who had non-institutional delivery, the prevalence rate of delayed breastfeeding initiation is 65%, which is much higher than for those who had institutional delivery. On the other hand, factors significantly associated with non-exclusive breastfeeding included children from different regions, place of residence, religion, caste, wealth index, number of ANC and mode of delivery (*p* < 0.001). The prevalence of non-exclusive breastfeeding among children in India is 36%, moreover, non-exclusive breastfeeding is 49% prevalent among mothers who did not visit antenatal care even for a single time. The prevalence of non-exclusive breastfeeding is 40% among children living in urban areas. Non-exclusive breastfeeding is prevalent in 39% of mothers who had preterm babies.

**Table 2:** Prevalence and association of delayed initiation of breastfeeding and non-exclusive breastfeeding with some selected background characteristics among children aged 0–23 months and 0–5 months, respectively, 2019-21

Variables	Delayed initiation of Breastfeeding		Non-exclusive Breastfeeding					
	Prevalence (%)	*P* value	0–1 month		2–3 months		4–5 months	
			Prevalence (%)	*P* value	Prevalence (%)	*P* value	Prevalence (%)	*P* value
**External environment factors**								
**Region**		< 0.001		p = 0.074		< 0.001		< 0.001
North	54.3		22.3		33		45.2	
Central	70.3		25.5		33.4		46.9	
East	56.3		23.7		34.9		57.6	
Northeast	45.6		27.2		37.3		53.1	
West	51.3		21.3		26.3		46.8	
South	44		27.9		32.2		55.1	
**Place of residence**		< 0.05		p = 0.380		p = 0.126		< 0.01
Urban	54.2		27.7		36.7		54.4	
Rural	57.8		23.5		31.7		49.5	
**Demographic & socioeconomic characteristics**								
**Maternal age at childbirth (years)**		< 0.001		p = 0.528		p = 0.060		< 0.05
Less than 20	54.8		23.7		34.8		53.7	
20–29	58		24.4		32.1		49.5	
30+	54.5		32.5		33.7		48.2	
**Religion**		< 0.001		< 0.001		< 0.001		< 0.001
Hindu	58.1		23.4		31.6		49.2	
Others	52.3		29.1		38.2		56.2	
**Caste of mother**		< 0.001		p = 0.118		< 0.01		< 0.01
SC	56.9		24.5		31.4		49.2	
ST	52.4		22.7		25.1		39.9	
OBC	59.8		24.5		32.9		51.2	
Others	53.3		25.4		38.3		55.9	
**Wealth index**		< 0.05		p = 0.469		p = 0.065		< 0.01
Poorest	58.7		23.8		32.4		49.1	
Poor	59.4		24.5		32.4		49.1	
Middle	55.8		22.7		30.4		50.5	
Richer	54.2		23.4		36.6		56.5	
Richest	55.4		29.7		33		49	
**Maternal education level**		< 0.001		p = 0.107		< 0.05		p = 0.200
No education	61.2		27.1		34.7		50.3	
Primary	57.4		25.2		32.5		55.2	
Secondary and above	55.7		23.6		32.6		50.1	
**Maternal mass-media exposure**		< 0.001		< 0.05		p = 0.084		p = 0.300
Not Exposed	61.6		26.1		34		52	
Exposed	55.1		23.8		32.5		50.2	
**Pregnancy & birth-related characteristics**								
**Desire of pregnancy**		< 0.05		p = 0.640		p = 0.203		p = 0.238
Wanted then	56.7		24.4		32.7		50.5	
Wanted later/ no more wanted	59.3		25.6		35.2		52.8	
**Preterm birth**		< 0.001		< 0.01		p = 0.889		< 0.05
No	56.3		23.7		32.7		50.3	
Yes	61.4		29.7		34.5		53.3	
**Sex of the child**		p = 0.483		p = 0.503		p = 0.073		< 0.05
Male	56.7		24.3		33.9		51.9	
Female	57.1		24.7		31.8		49.5	
**Maternal health service utilization**								
**Number of ANC**		< 0.001		< 0.001		< 0.001		< 0.001
4 + times	52.1		22.4		31.2		49	
1–3 times	63.6		26.4		33.6		50.6	
None	61.6		33.8		44.2		64.9	
**Place of delivery**		p = 0.626		< 0.001		< 0.05		< 0.001
Institutional delivery	56.1		23.9		32.8		50.1	
Delivery at home	64.6		30.5		34.3		56.6	
**Mode of delivery**		< 0.001		p = 0.673		< 0.01		< 0.05
Non-Caesarean section	54.9		24.3		31.9		49.4	
Caesarean section	63.5		25.1		36.1		54.5	
**Postnatal care services**		< 0.001		< 0.001		< 0.001		< 0.001
No check	64.9		29.7		36.6		54.3	
Doctor	53.5		23.3		32.6		50.9	
Nurse/Midwife	57.8		23.2		31.1		48.5	
Non-skilled provider	60		26.4		35.5		49.6	
**Total**	**56.9**		**24.5**		**32.9**		**50.7**	

### Determinants of delayed initiation of breastfeeding and non-exclusive breastfeeding

Tables 3 and 4 shows the findings of the logistic regression models for both outcome variables. Factors significantly associated with increased odds of delayed initiation of breastfeeding included infants from in the central region (OR 2.19; 95% CI 2.09, 2.29), mothers belong to the 20 to 29 years age group at the time of childbirth (OR 1.02; 95% CI 0.98, 1.05), caesarean deliveries (OR 1.97; 95% CI 1.90, 2.05), and for those who had less than nine months of pregnancy (OR 1.15; 95% CI 1.10, 1.20). An increased odds for delayed breastfeeding was also associated with poor household wealth status, in the case of non-institutional deliveries occurred under untrained persons (OR 1.02; 95% CI 0.97, 1.08). For factors associated with non-exclusive breastfeeding, the odds significantly increased among children belonging to the richest household status (OR 1.30; 95% CI 1.17, 1.45), mothers who had less than nine months of pregnancy period (OR 1.15; 95% CI 1.06, 1.25), mother gave birth in non-health facility (OR 1.17; 95% CI 1.05, 1.31), caesarean deliveries (OR 1.15; 95% CI 1.07, 1.24). The odds were significantly lower among children whose mothers were exposed to media (OR 0.87; 95% CI 0.81, 0.93) and those who availed postnatal care from skilled medical professionals such as doctors (OR 0.84; 95% CI 0.77, 0.92) and nurses (OR 0.81; 95% CI 0.74, 0.88).

**Table 3:** Demographic, socioeconomic, pregnancy and birth-related characteristics and maternal health service utilization determinants of children aged 0–23 months in India, 2019-21

Variables	Delayed initiation of breastfeeding			
	Unadjusted		Adjusted	
	OR	95% CI	OR	95% CI
**External environment factors**				
**Region**				
North ®				
Central	2.11***	2.02,2.20	2.12***	2.03,2.22
East	1.34***	1.29,1.40	1.29***	1.22,1.35
Northeast	0.67***	0.64,0.70	0.63***	0.59,0.66
West	1.11***	1.05,1.18	1.15***	1.09,1.22
South	0.89***	0.85,0.94	0.80***	0.76,0.84
**Place of residence**				
Urban ®				
Rural	1.05**	1.02,1.09		
**Demographic & socioeconomic characteristics**				
**Maternal age of childbirth (years)**				
Less than 20 ®				
20–29	1.07***	1.03,1.10	1.03	0.99,1.06
30+	0.90**	0.83,0.97	0.92*	0.85,1.00
**Religion of mother**				
Hindu ®				
Others	0.65***	0.63,0.67		
**Caste of mother**				
SC	1.07***	1.03,1.12		
ST	0.72***	0.68,0.75		
OBC	1.23***	1.18,1.28		
Others ®				
**Wealth index**				
Poorest ®				
Poor	1	0.96,1.04	1.08***	1.04,1.13
Middle	0.97	0.93,1.01	1.08**	1.03,1.13
Richer	0.94**	0.90,0.98	1.02	0.97,1.07
Richest	0.95*	0.91,1.00	0.99	0.94,1.05
**Maternal education level**				
No education®				
Primary	0.89***	0.84,0.93		
Secondary and above	0.88***	0.85,0.91		
**Maternal mass-media exposure**				
Not Exposed ®				
Exposed	0.85***	0.83,0.88	0.92***	0.89,0.95
**Pregnancy & birth-related characteristics**				
**Desire of pregnancy**				
Wanted then ®				
Wanted later/ no more wanted	1.07**	1.02,1.13		
**Duration of pregnancy**				
Nine months and above®				
Less than nine months	1.18***	1.13,1.23	1.14***	1.09,1.19
**Sex of the child**				
Female ®				
Male	1.01	0.98,1.03		
**Maternal health service utilization**				
**Number of ANC**				
4 + times ®				
1–3 times	1.49***	1.45,1.54	1.43***	1.38,1.47
none	1.25***	1.19,1.31	1.28***	1.22,1.35
**Place of delivery**				
Institutional delivery ®				
Delivery at home	1.01	0.97,1.05		
**Mode of delivery**				
Non-Caesarean section ®				
Caesarean section	1.59***	1.54,1.65	2.02***	1.94,2.10
**Postnatal care services**				
No check ®				
Doctor	0.84***	0.81,0.87	0.82***	0.78,0.86
Nurse/Midwife	0.93***	0.89,0.96	0.78***	0.75,0.82
Non-skilled provider	0.88**	0.81,0.96	0.76***	0.70,0.83

Note- ref: Reference category; level of significance: *p < 0.10, **p < 0.05, ***p < 0.01

*Adjusted for region, maternal age of childbirth, wealth index, maternal mass-media exposure, duration of pregnancy, place of delivery, mode of delivery, postnatal care services*

**Table 4:** Demographic, socioeconomic, pregnancy and birth-related characteristics and maternal health service utilization determinants of children aged 0–5 months in India, 2019-21

Variables	Non-exclusive breastfeeding											
	0–1 month				2–3 months				4–5 months			
	Unadjusted		Adjusted		Unadjusted		Adjusted		Unadjusted		Adjusted	
	**OR**	**95% CI**	**OR**	**95% CI**	**OR**	**95% CI**	**OR**	**95% CI**	**OR**	**95% CI**	**OR**	**95% CI**
**External environment factors**												
**Region**												
North ®												
Central	0.99	0.84,1.16	0.99	0.84,1.17	0.89	0.78,1.03	0.93	0.80,1.07	0.89	0.78,1.01	0.94	0.82,1.08
East	0.93	0.78,1.11	0.9	0.75,1.09	0.96	0.82,1.11	0.95	0.80,1.12	1.16*	1.01,1.35	1.20*	1.02,1.40
Northeast	1.17	0.97,1.40	1.11	0.91,1.35	1.48***	1.26,1.74	1.44***	1.21,1.71	1.57***	1.34,1.84	1.55***	1.30,1.85
West	0.94	0.75,1.17	1.01	0.80,1.27	0.82	0.66,1.01	0.81	0.66,1.01	1.05	0.86,1.28	1.07	0.87,1.30
South	1.18	0.97,1.44	1.29*	1.05,1.59	0.84	0.71,1.00	0.81*	0.67,0.97	1.36***	1.16,1.60	1.37***	1.16,1.62
**Place of residence**												
Urban ®												
Rural	0.94	0.82,1.08			0.91	0.81,1.03			0.84**	0.75,0.94		
**Demographic & socioeconomic characteristics**												
**Maternal age of childbirth (years)**												
Less than 20 ®												
20–29	0.95	0.84,1.07	0.97	0.85,1.09	0.88*	0.79,0.98	0.88*	0.78,0.98	0.89*	0.81,0.98	0.9	0.81,1.00
30+	0.87	0.64,1.17	0.81	0.60,1.11	0.87	0.67,1.13	0.8	0.61,1.04	1.07	0.84,1.36	0.95	0.74,1.22
**Religion of mother**												
Hindu ®												
Others	1.36***	1.21,1.53			1.59***	1.43,1.76			1.44***	1.30,1.60		
**Caste of mother**												
SC	0.92	0.78,1.09			0.78**	0.68,0.91			0.82**	0.71,0.94		
ST	0.88	0.74,1.04			0.79**	0.68,0.92			0.79**	0.69,0.91		
OBC	0.84*	0.72,0.97			0.77***	0.68,0.88			0.82**	0.72,0.93		
Others ®												
**Wealth index**												
Poorest ®												
Poor	1	0.86,1.16	1.09	0.93,1.28	1.02	0.89,1.17	1.1	0.95,1.28	1.03	0.90,1.17	1.11	0.97,1.27
Middle	1	0.85,1.17	1.14	0.95,1.36	0.97	0.84,1.12	1.13	0.97,1.33	0.95	0.83,1.08	1.06	0.91,1.23
Richer	0.94	0.79,1.11	1.13	0.92,1.38	1.19*	1.03,1.37	1.44***	1.22,1.71	1.28***	1.12,1.47	1.48***	1.25,1.74
Richest	1.14	0.95,1.36	1.41**	1.13,1.76	0.99	0.84,1.16	1.21	1.00,1.47	1.03	0.89,1.19	1.26*	1.06,1.51
**Maternal education level**												
No education®												
Primary	1.05	0.86,1.27			0.92	0.77,1.10			1.07	0.91,1.26		
Secondary and above	0.9	0.79,1.03			0.85*	0.75,0.96			0.95	0.85,1.07		
**Maternal mass-media exposure**												
Not Exposed ®												
Exposed	0.86*	0.77,0.97	0.85*	0.74,0.98	0.91	0.82,1.01	0.89	0.79,1.01	0.95	0.86,1.05	0.9	0.80,1.01
**Pregnancy & birth-related characteristics**												
**Desire of pregnancy**												
Wanted then ®												
Wanted later/ no more wanted	1.05	0.87,1.27			1.12	0.94,1.32			1.1	0.94,1.30		
**Duration of pregnancy**												
Nine months and above®												
Less than nine months	1.30***	1.11,1.52	1.31***	1.12,1.53	1.01	0.88,1.17	1.01	0.87,1.16	1.15*	1.00,1.31	1.17*	1.02,1.34
**Sex of the Child**												
Female ®												
Male	0.96	0.87,1.07			1.09	0.99,1.20			1.10*	1.01,1.21		
**Maternal health service utilization**												
**Number of ANC**												
4 + times ®												
1–3 times	1.18**	1.06,1.33	1.18**	1.05,1.33	1.14*	1.03,1.27	1.12*	1.01,1.25	1.03	0.93,1.13	1.06	0.96,1.17
none	1.89***	1.56,2.29	1.70***	1.39,2.08	1.87***	1.57,2.22	1.71***	1.42,2.05	1.74***	1.46,2.08	1.61***	1.34,1.93
**Place of delivery**												
Institutional delivery ®												
Delivery at home	1.46***	1.25,1.70	1.25*	1.03,1.53	1.20*	1.04,1.40	0.93	0.77,1.13	1.32***	1.15,1.52	1.23*	1.03,1.48
**Mode of delivery**												
Non-Caesarean section ®												
Caesarean section	1.03	0.90,1.18	1.06	0.92,1.23	1.16**	1.04,1.30	1.24***	1.09,1.40	1.15*	1.03,1.28	1.11	0.98,1.25
**Postnatal care services**												
No check ®												
Doctor	0.71***	0.62,0.82	0.82*	0.69,0.97	0.78***	0.69,0.89	0.86	0.73,1.00	0.84**	0.75,0.95	0.96	0.83,1.11
Nurse/Midwife	0.67***	0.57,0.77	0.80*	0.67,0.95	0.72***	0.63,0.83	0.81**	0.69,0.95	0.71***	0.62,0.81	0.91	0.78,1.06
Non-skilled provider	0.8	0.59,1.09	0.83	0.61,1.13	0.96	0.72,1.27	1.1	0.82,1.48	0.8	0.61,1.04	0.87	0.66,1.14

Note- ref: Reference category; level of significance: *p < 0.10, **p < 0.05, ***p < 0.01

*Adjusted for region, maternal age of childbirth, wealth index, maternal mass-media exposure, duration of pregnancy, place of delivery, mode of delivery, postnatal care services*

## Discussion

This paper explores the association between socioeconomic, environmental, pregnancy and birthing characteristics, maternal healthcare services determinants with delayed initiation of breastfeeding and non-exclusive breastfeeding. Infants residing in rural areas, who belong to poor wealth status, whose mothers had caesarean deliveries, as well as experienced non-institutional deliveries by non-health professionals, not exposed to mass-media, preterm births, and who received fewer or no prenatal and postpartum check-ups were more likely to suffer delayed breastfeeding. On the other hand, infants from rich household, who reside in urban areas, whose mothers had caesarean deliveries, home childbirth and none or less access to antenatal and postnatal services by health professionals have an increased likelihood of non-exclusive breastfeeding. In contrast, the results reveal that characteristics such as place of residence, maternal age at childbirth, maternal educational status, exposure to mass media, and gender of the child were not significantly associated with non-exclusive breastfeeding. This is in line with a study from Tanzania, where none of the factors was associated with EBF, as exclusive breastfeeding was not a traditional practice in Tanzania [25]. However, in India, traditional infant feeding practices in the community, such as giving water and other water-based fluids to infants before six months of age, are practiced at the community level [26, 27]. Beside these practices, a lack of awareness, and social norms have led to a lower prevalence of EBF than the recommended levels. This highlights the necessity of, awareness programs and interventions are necessary to increase knowledge and promote the benefits of EBF among mothers, families, and communities.

This study indicated that, in comparison to the Northern region of India, the likelihood of delayed breastfeeding initiation was lowest in the North-East, followed by South, East, and Western regions. Contrarily, compared to the Northern region, the odds of delayed initiation of breastfeeding were higher in the Central region, which is in line with several other research [28, 29]. In earlier literatures, the root causes of regional disparities in delayed breastfeeding patterns have not been adequately explained. So far, there may be other reasons to consider, such as regional cultural beliefs that discourage the use of mother’s first milk [30]. Despite knowing that colostrum is advantageous and guards the newborn from diseases, elder mothers in some parts of India believe that colostrum is harmful to the infant, and such recommendations from mothers-in-law do not support the ideal breastfeeding approaches for child [31]. In addition to this, some studies have revealed that central region states have substantially less access to and usage of maternal healthcare services than other areas, such as the north, east, and south [32, 33]. Just 11.3% of pregnant women in India’s central region received adequate antenatal care services throughout their gestation period, and mothers often lack knowledge about the benefits of starting breastfeeding early which might be lead to delayed initiation of breastfeeding [34]. Region-specific policies and interventions that target women in their immediate community are need to be implemented in India.

Among the socioeconomic factors, a lower wealth index was associated with delayed initiation of breastfeeding, which is consistent with some previous studies [24, 35]. Interventions to raise awareness of the significance of early initiation of breastfeeding should target women who are economically underprivileged. This might be a possible explanation that in India, lower wealth quintile women may have lower levels of education, limited access to antenatal and postnatal services, and less awareness about the importance of early initiation of breastfeeding. They may have less knowledge about the benefits of breastfeeding for both the mother and the infant, and may not receive adequate education or counselling on breastfeeding practices during pregnancy and after childbirth. A study by Ketbi et. al (2018) have also similar findings where women with lower level of education and low family income had poor knowledge of breastfeeding practices [36]. Cultural and traditional beliefs hinder early imitation of breastfeeding, a primary study from rural India reported the local belief that “mother’s milk is ‘not ready’ until two-to-three days postpartum” [30].

As reported in a previous study, our analysis found that mothers had experienced preterm childbirth were more likely to delayed initiation of breastfeeding due to probable health complications of the newborn [37]. This might result in infant mortality and other nutritional deficiency among children.

The results of our analysis found that compared to mothers who gave birth vaginally, mothers who underwent a caesarean section experienced a considerable delay in initiating to breastfeed, which also has been discussed in previous studies in developing nations [38, 39]. Early breastfeeding was challenging in certain hospitals because newborns can be placed in separate rooms so that mothers can rest following surgery. After caesarean births, procedures avoiding intimate contact between mothers and babies should be improved [40].

The likelihood of postponing the initiation of breastfeeding was shown to be higher among births delivered at home or in any other non-health facility. Traditional birth attendants and home births are still frequently practiced, especially in rural parts of developing nations [41, 42]. Support from professional health attendants to encourage mothers to breastfeed the newborn immediately after delivery will help the child to overcome potential barriers.

From the results, we found that newborns from rich households were more likely to breastfed non-exclusively which is similar to some previous studies [29, 35]. This unsatisfactory breastfeeding habit may be explained by the frequent exposure to different varieties of infant formula feeding and the financial capacity to afford the formula feeding. Strategies to encourage exclusive breastfeeding among mothers with higher household wealth indexes are necessary. Infants delivered by caesarean section and women who choose non-institutional delivery methods are far more likely to get complementary feedings.

The strength of this study includes use of a nationally representative survey with a large sample size that provided adequate power to include various potential factors in the analysis. The NFHS uses standardized questionnaires and methodology which facilitate inter-country comparison. The findings of this study will be useful in developing a suitable management strategy, highlighting any areas of exclusive breastfeeding promotions that need to be addressed and will also help in the effective promotion and implementation of government intervention programs such as ‘Mothers Absolute Affection’ (MAA) to the targeted groups and communities.

However, this study also met with few limitations. Firstly, the cross-sectional nature of dataset does not allow for any causal inference. Secondly, as recommended by the WHO, the assessment of exclusive breastfeeding was solely based on the 24-hour recall data, which might have underestimated the percentage of babies who were not exclusively breastfed [11]. However, it is unlikely that these restrictions will compromise the reliability of the findings.

## Conclusions

Infants from low-income households, from rural areas, mothers who underwent caesarean sections, had preterm deliveries, gave birth at home, and women who did not get postpartum care services are at risk of delayed initiation of breastfeeding and non-exclusive breastfeeding. The relationships between several categories of factors and non-exclusive breastfeeding and delayed initiation of breastfeeding highlight the necessity of conducting comprehensive public health strategies utilizing a multi-sectoral strategy to encourage breastfeeding behaviours in India. It should be a primary concern to raise knowledge of the benefits of early initiation and exclusive breastfeeding among women and families, even those from affluent households. The Sustainable Development Goals, particularly Goal 3 on ensuring everyone’s health and well-being, will be more successfully attained as a result of improved breastfeeding practices among infants aged 0–23 months.

## Data Availability

The data used in this study is available at request at https://www.dhsprogram.com.

## Abbreviations

CI: Confidence Interval
EBF: Exclusive Breastfeeding
MAA: Mothers Absolute Affection
NFHS: National Family Health Survey
OR: Odds Ratio
UNICEF: United Nations Children’s Fund
WHO: World Health Organization
